# Effectiveness and cost of recruitment strategies for a community-based randomised controlled trial among rainwater drinkers

**DOI:** 10.1186/1471-2288-9-51

**Published:** 2009-07-16

**Authors:** Shelly Rodrigo, Martha Sinclair, David Cunliffe, Karin Leder

**Affiliations:** 1 Department of Epidemiology and Preventive Medicine, School of Public Health and Preventive Medicine, Monash University, Melbourne, Australia; 2 Department of Health, South Australia

## Abstract

**Background:**

Community-based recruitment is challenging particularly if the sampling frame is not easily defined as in the case of people who drink rainwater. Strategies for contacting participants must be carefully considered to maximise generalisability and minimise bias of the results. This paper assesses the recruitment strategies for a 1-year double-blinded randomised trial on drinking untreated rainwater. The effectiveness of the recruitment strategies and associated costs are described.

**Methods:**

Community recruitment of households from Adelaide, Australia occurred from February to July 2007 using four methods: electoral roll mail-out, approaches to schools and community groups, newspaper advertising, and other media involvement. Word of mouth communication was also assessed.

**Results:**

A total of 810 callers were screened, with 53.5% eligible. Of those who were eligible and sent further information, 76.7% were willing to participate in the study and 75.1% were enrolled. The target for recruitment was 300 households, and this was achieved. The mail-out was the most effective method with respect to number of households randomised, while recruitment via schools had the highest yield (57.3%) and was the most cost effective when considering cost per household randomised (AUD$147.20). Yield and cost effectiveness were lowest for media advertising.

**Conclusion:**

The use of electoral roll mail-out and advertising via schools were effective in reaching households using untreated rainwater for drinking. Employing multiple strategies enabled success in achieving the recruitment target. In countries where electoral roll extracts are available to researchers, this method is likely to have a high yield for recruitment into community-based epidemiological studies.

## Background

Recruiting participants is a critical stage in any research process and whether or not recruitment targets are achieved may depend on the strategies used. In clinical settings, participants are often recruited through their medical providers, while in community-based studies recruitment samples the general population. Challenges to community-based recruitment include the location and size of the eligible target population, especially if it is a small subset of the total population. Recruitment may be even more complex if specific criteria are required for eligibility, and if the sampling frame is not easily defined as in the case of the use of alternative water sources. Recruitment of units, such as couples and families, rather than individuals also poses problems for researchers as the decision to participate is not unilateral. The strategies for contacting participants need to be tailored to each scenario and must be carefully considered to maximise generalisability and minimise bias of the results.

Current climate changes globally combined with high evaporation rates have resulted in a decrease in surface and ground water sources, resulting in the progressive need for alternative supplies of drinking water [1]. Many developing countries currently use rainwater as a drinking water source because mains water supplies are not sufficiently developed to serve the entire country or due to ground water contamination [2]. However, in Australia, although 19% of households have rainwater tanks [3], some health authorities do not endorse the consumption of untreated rainwater if an alternative tap water supply is available [4,5]. In New Zealand, health authorities recommend suitable treatment of the collected rainwater if it is to be used for drinking, but only 10% of the population use rainwater supply for drinking [6-8].

A randomised controlled trial was set up to determine the health risk of drinking untreated rainwater by recruiting 300 households in metropolitan Adelaide, Australia. This paper describes the recruitment strategies employed to enlist household participation in the trial. The associated costs for the recruitment strategies employed are also described.

## Methods

### Study design and study population

Households were recruited between February and June 2007 from metropolitan Adelaide and surrounding urban regions of South Australia. Participating households were required to have no less than 4 members with at least two aged 1 – 15 years, and needed to drink untreated rainwater from an above-ground tank. Participants were also required to own their home or have a rental history of 12 months or more. Exclusion criteria included connection of the rainwater tank to a mains water system, the use of disinfection or filters attached to the tank outlet, routine boiling of rainwater prior to drinking or an immunocompromised status. Households were randomised to receive either a real or a sham water treatment unit.

The randomised controlled trial was approved by Monash Standing Committee on Ethics in Research Involving Humans (SCERH) and South Australia Department of Health Human Research Ethics Committee. A sample size of 300 households was required in order to enable detection of a 25% reduction in the overall rate of gastroenteritis in the experimental group with 80% power using a two-sided 5% significance level. Written consent to participate in the study was obtained from all household members over the age of 18 and study participants were required to complete health diaries for a 12 month period

### Recruitment strategies

The recruitment strategies used included mail-out of invitation letters using an electoral roll extract; distribution of pamphlets in schools and school newsletter inserts approximately one month after pamphlet distribution; community promotion (pamphlets and posters on local notice boards); advertisements in the local newspaper and other media interventions (articles in newspapers and on the internet, and discussion on local "talk" radio programs). Other methods of hearing about the study were determined at the first interview, resulting in an additional category of family/friend (word of mouth communication) being obtained. Two of these strategies namely school newsletter inserts and other media interventions, as well as family/friend communication involved no direct costs. For all methods of recruitment, the text contained the same information.

The target area was subdivided into sections, based on the presumed distribution of rainwater tanks, and a sequential distribution of letters, pamphlets and posters was implemented. A total of 150, 308 invitation letters were mailed to households during the period February to May 2007, with concurrent distribution of pamphlets in schools (29,728) and posters on community boards (130). An extract of voters aged 18 – 49 years was obtained from the Australian Electoral Commission (AEC) and filtered by suburb, postcode, street number and address to produce lists of unique households in the target area for mailing. Schools in the target area were identified using data from the Department of Education and Children's Services, South Australia and pamphlets were sent to teachers and principals to be distributed to children in childcare, kindergarten, primary and the lower levels of high schools. During the period April – June 2007, four advertisements were placed in the community and metropolitan (Saturday edition) newspapers, the latter having a readership of 691,000 [8]. Radio interviews were conducted in May 2007.

**Figure 1:**
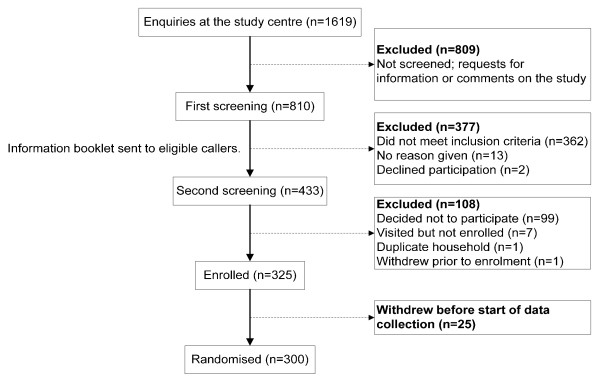
**Flow chart of participant recruitment in the Adelaide Rainwater Study**.

The content of all methods of recruitment were the same – the eligibility criteria and an invitation for eligible participants to call a toll free number, however more detail was added to the letters and pamphlets. At time of the call, households were screened for eligibility using a simple telephone questionnaire which addressed tank type, location of household, the use of filter devices or disinfection, and the number and ages of persons in the house. At this first screening, callers were asked how they initially heard about the study and if there was any other method by which they obtained information about the study. An information booklet was then sent to households that were interested and eligible to participate. Study personnel contacted households by telephone one week after the booklets were sent and a second questionnaire was administered to confirm eligibility and willingness to participate, and to arrange for an enrolment visit to obtain informed consent and baseline data. A flowchart of the recruitment process is presented in Figure 1.

### Analysis

The recruitment method which yielded the most eligible participants at each stage of the process was determined by querying the database for the recruitment source of each caller, their eligibility status and whether they were ultimately enrolled into the study. Data were analysed for effectiveness of the recruitment method in three ways: percent eligible at a particular stage of recruitment, the percent yield (the number of households randomised as a proportion of the total callers reporting the strategy), and the cost per household randomised. The last two measures were used to determine which strategy was the highest yielding and most cost effective for recruitment of participants into the data collection phase. Cost effectiveness was determined by dividing the total cost of any method by the number of households randomised as a result of that method. Total cost was determined for each method by considering material and personnel costs. Invitation letters were generated by a mailing house hence the figure given includes all associated costs (printing, envelopes, inserting letter in envelopes, postage) as well as the cost of obtaining the AEC data extract. For schools and community methods, the total cost includes cost of posters/pamphlets and delivery by personnel to the locations. No costs were incurred for school newsletter inserts where brief text about the study was included in regular print or electronic newsletters sent by the school.

## Results

A total of 1619 calls were received by the study centre during the recruitment period. Of these 809 were from callers who only wished to get information or make comments about the study and hence were not screened by study personnel. Figure 1 gives details on the 810 callers who were screened, of whom 53.5% (433) met the eligibility criteria and were willing to receive further information, and shows the flow of the participants at each stage of the recruitment process. Of those who were eligible and sent further information, 76.7% (332) were willing to participate in the study and 75.1% (325) were enrolled. Overall, 40.1% (325) of initially screened callers were ultimately enrolled and 37% (300) randomised. The effectiveness of each recruitment strategy is shown in Table 1.

**Table 1:** Effectiveness of recruitment strategies

	Stages of recruitment/number (%)^#^				
	
		Households eligible at:			Start of study
			
Strategy	All callers*	First Screen	Second Screen	Enrolment	
	(n = 810)	(n = 433)	(n = 332)	(n = 325)	(n = 300)
Invitation letter	446 (55.1)	266 (61.4)	203 (61.1)	198 (60.9)	182 (60.7)
School	89 (11.0)	71 (16.4)	57 (17.2)	56 (17.2)	51 (17.0)
Newspaper advertising	43 (5.3)	27 (6.2)	18 (5.4)	18 (5.5)	17 (5.7)
Community	29 (3.6)	16 (3.7)	12 (3.6)	12 (3.7)	12 (4.0)
Other media	74 (9.1)	43 (9.9)	33 (9.9)	32 (9.8)	30 (10.0)
Family/Friend	18 (2.2)	10 (2.3)	9 (2.7)	9 (2.8)	8 (2.7)

^# ^Percent recruitment at any stage = 100 (number of callers for the method/total number of callers at the specific stage)

*The method of recruitment was missing for 111 (13.7%) of the initial callers.

For the mail-out, a total 150,308 letters were sent, resulting in 446 initial enquiries. Of the recruitment methods used, the mail-out resulted in 55.1% of the total calls screened and 61.4% (266) of contacts amongst eligible households. With respect to the number of households randomised, the mail-out was the most effective method with 60.7% of enrolled households reporting that they first heard of the study via the invitation letter.

Participants were asked whether they heard about the study from any other source. Of the 699 initial callers who gave details on the first method of communication, 83.7% (585) heard about the study from the primary method only. Table 2 shows the percent distribution of all callers and those who started the study and heard of the study via only one method. A total of 106 (15.2%) callers heard about the study via one other method, seven heard about the study via two other methods and one caller heard about the study via three other methods. No data were obtained on what method prompted these callers to contact the study centre.

**Table 2:** Distribution of callers who heard about the study via a single strategy

	All callers*		Start of Study	
		
	No.	No. (%)	No.	No. (%)
Invitation letter	446	404 (90.6)	182	150 (82.4)
School	89	57 (64.0)	51	29 (56.9)
Newspaper advertising	43	37 (86.0)	17	13 (76.5)
Community	29	20 (69.0)	12	5 (41.7)
Other media	74	55 (74.3)	30	17 (56.7)
Family/Friend	18	12 (66.7)	8	4 (50.0)
All strategies	699	585 (83.7)	300	218 (72.7)

*The method of recruitment was missing for 111 (13.7%) of the initial callers.

Table 3 shows the yield and cost effectiveness of each method for recruitment of households into the study. While only 17% (51) of households randomised were recruited via schools, this method had the highest yield (57.3%) and was the most cost effective when considering the number of households randomised (AUD$147.20). Yield was lowest for media advertising which was also the least cost effective method per household randomised.

**Table 3:** Yield and cost effectiveness of methods for recruitment into the study

Strategy	Percent yield^1^	Total cost^2^	Cost per household randomised^3^
*Direct costs*			
Invitation letter	40.8	$ 31,534.22	$ 173.26
School	57.3	$ 7,507.00	$ 147.20
Newspaper advertising	39.5	$ 5,768.28	$ 339.31
Community	42.9	$ 2,736.40	$ 228.03
*No direct costs*			
Other media	40.5	$ -	$ -
Family/Friend	44.4	$ -	$ -

^1^Percent yield = 100 * (number of households randomised/total number of callers reporting the strategy)

^2^Total cost includes material and personnel cost for all methods.

^3^The 809 unscreened calls are not included in these calculations as it is not possible to attribute these calls to a specific recruitment strategy.

## Discussion

The intention of this paper is to describe the strategies found to be successful in recruiting participants into a community-based randomised controlled trial investigating the use of an alternative drinking water source. This information is important and valuable as it can be applied in other situations where recruitment involves a niche population group with specific eligibility requirements.

One of the major challenges of this study was the identification of appropriate study participants for recruitment from within the larger community. The target for enrolment was those households drinking untreated rainwater from an above-ground rainwater tank, and the identification of such households was impossible since no listing of the sampling pool exists and only an estimated 10.6% of people in Adelaide drink rainwater [3]. Additionally, households with rainwater tank supplies may not be adjacent to each other or even in neighbouring areas. Application of the inclusion criteria and the requirement for above-ground and not underground rainwater tanks further decreased the potential sample pool by an unknown percent. Since the target group for recruitment may be a small percent of the total population, a general and broad-based approach to recruitment was needed. The selected target population was within the large area encompassing metropolitan Adelaide and the western bordering region and contained approximately 200,000 households [9].

Poor practices during recruitment, such as enrolment of potential participants who show borderline interest, can result in low retention rates in longitudinal studies. A non-aggressive recruitment approach was therefore implemented in this study in order to ensure that the rate of dropout during the 12 months of data collection was minimized. Recruitment via invitation letters based on the electoral roll extract with a requirement that interested participants actively contact the study centre was one way of ensuring significant interest among those enrolled. A major limitation of such an approach is that it restricts the generalisability of the results and increases selection bias with only the most motivated individuals responding to any invitation.

Recruitment through the use of the mail-out was undertaken in our study to reach as many potential participants as possible and was the most effective method in terms of the number of households randomised. However, one disadvantage of a large mail-out is that generic letters are impersonal and of varying relevance to individual recipients. Considering that 150,308 letters were sent, the overall response rate to the mail-out was only 0.3% if the total numbers of letters sent is used as the denominator. However, since only about 10% of households drink rainwater, the response rate among potentially eligible households increases to 3%. When demographic data are also considered, the requirement of ≥4 household members with at least 2 children suggests that a response rate of approximately 24% from households meeting all inclusion criteria was obtained. When the number of pamphlets distributed in schools was considered, the overall response rate was also 0.3%.

Advertising as a recruitment method can potentially reach large sectors of the community. Advertisements were placed on several occasions in the Saturday edition of the local newspaper and in community papers. The Saturday readership for the local newspaper was 691,000 readers for the period June 2006 to June 2007 [10]. Following the publication of the advertisements, the study centre received a rush of phone calls, many of which were from obviously ineligible callers who wanted to comment on the study rather than enquire about participation. The overall contribution and yield from advertising was lower than the electoral roll mail-out and other media methods.

School-based advertising was considered appropriate since the target group were required to have households with at least 2 children under the age of 15. Consequently kindergartens, primary schools, and students up to the lower levels of high school were provided with information on the study. This strategy proved to have a high yield, presumably because it targeted recruitment to populations more likely to fit with required demographic inclusion criteria. However this method may not be applicable to studies in which eligibility does not involve school-aged children as part of the study group.

In conducting other community-based studies some researchers have commented on the importance of involving members of the target community to assist with recruitment [11]. However this was not possible in our study since there was no clearly defined community group. Community involvement may therefore not always be practical or a guarantee of increased participation in studies, and in our study community recruitment methods generated only a small number of eligible households compared with other approaches.

The data showed that the majority of the initial callers (83.7%) and study participating households (72.7%) heard about the study through a single contact method. Exposure to the recruitment strategy can be concluded as being the method which prompted calls to the study centre to express interest. In the case of the 115 initial callers who called after exposure to two or more recruitment strategies, the initial exposure is not an adequate proxy measure for determining which strategy caused them to call. It may be that the initial exposure generated interest and a desire to call while the second exposure acted as a reminder which resulted in calls to the study centre.

When cost per household randomised was considered, the most and least cost-effective methods were schools and newspaper advertising, respectively. The average cost per participant enrolled using the electoral roll mail-out was approximately 49% less than the cost for recruitment using advertising. This is contradictory to the findings of Bjornson-Benson et al. [12] who reported that media methods were most cost-effective in recruitment. However, in their study the authors grouped all media methods into one category for analysis. Separation of the media category into paid advertising versus unpaid, newspaper articles and radio talk programs gives a better indication of the actual costs incurred per participant recruited. While the total expenditure of advertising was low in comparison with that associated with a mass mailing, the latter strategy was the most frequent information source in our study.

The agreement and hence willingness to participate in health related studies is dependent on many factors. After the screening phases, 75.1% of the eligible callers agreed to participate. This compares favourably with another similar study, the Melbourne Water Quality Study, where 60.7% of eligible callers agreed to participate in a tap water study of similar design [13]. In our current study, participation may have been facilitated by the high awareness of increasing water shortages in Australia. Presumably those with a high degree of interest in water and environmental issues were more likely to contact the study to express interest in participation.

## Conclusion

Assessment of the effectiveness of the recruitment strategies by households screened and enrolled into a study examining health effects of drinking untreated versus treated rainwater showed that use of the electoral roll mail-out and enrolment via schools were both effective methods in reaching the required niche population. Although multiple recruitment strategies have the potential to aid in achieving recruitment targets, in countries where an electoral roll extract is available to researchers, this method is likely to have a high yield for recruitment into community-based epidemiological studies. Tailoring the recruitment strategies based on the eligibility criteria may also be advantageous. Indeed, in our study, the ability to target potentially eligible participants via schools meant that this method was also both successful and low-cost. A limitation of the study is that the participants were not asked what method of contact prompted them to call the study centre. It may be that exposure to other forms of information may have prompted some participants to make contact. This type of information has the potential to assist with streamlining recruitment strategies for community-based epidemiological studies.

## Competing interests

The authors declare that they have no competing interests.

## Authors' contributions

SR analysed the data and drafted the manuscript. MS and KL were involved in the design and management of the study, and reviewed the manuscript. DC participated in the study design and management of recruitment. All authors had full access to data in the study and have read and approved the final manuscript.

## Pre-publication history

The pre-publication history for this paper can be accessed here:

http://www.biomedcentral.com/1471-2288/9/51/prepub
